# Complete acute uterine inversion

**DOI:** 10.11604/pamj.2013.16.33.1956

**Published:** 2013-09-30

**Authors:** Lawrence Mbuagbaw, Patrick Mbah Okwen

**Affiliations:** 1 Centre for the Development of Best Practices in Health (CDBPH), Yaoundé Central Hospital, Avenue Henri Dunant, Messa, PO Box 87, Yaoundé, Cameroon

**Keywords:** Acute uterine inversion, post-partum, placenta, childbirth

## Image in medicine

Acute uterine inversion is a rare and life-threatening post-partum complication which often occurs when the placenta fails to detach from the uterus after childbirth. The uterine fundus falls into the endometrial cavity and may descend to the cervix (incomplete) or beyond the cervix (complete). Death may occur in 15% of the affected mothers due to pain, blood loss and shock. The shock is often described as being “out of proportion” to the bleeding. Uterine inversion is associated with primiparity, the use of oxytocin, macrosomia and fundal insertion of the placenta. Premature traction on the umbilical cord and fundal pressure before placental separation are the usual direct causes. Care for acute uterine inversion involves pain management, resuscitation and replacement of the inverted uterus before oedema sets in. Surgery may be required in severe cases. We present here the case of a 37 year old woman, gravida 4 para 4, who was rushed into our services. This is a picture of complete uterine prolapse with a totally inverted endometrium. The entire uterus can be seen protruding through the vulva. The placenta is no longer attached, but its point of insertion can be seen (dark red central portion). She was in pain, but was not bleeding at the time of admission. There were no signs of shock. Management involved parenteral antibiotics and fluids; cleaning and debridement of exposed endometrium; and replacement of the uterus in the abdominal cavity using the Johnson manoeuvre.

**Figure 1:**
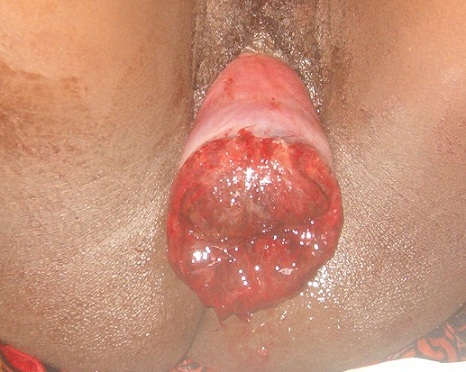
Completely inverted uterus

